# Primary lymphoma of the head and neck: two case reports and review of the literature

**DOI:** 10.1186/1757-1626-1-426

**Published:** 2008-12-30

**Authors:** Ismail Essadi, Nabil Ismaili, Elmehdi Tazi, Sanaa Elmajjaoui, Ammar Saidi, Mohammed Ichou, Hassan Errihani

**Affiliations:** 1 Department of Medical Oncology, National Institute of Oncology, Rabat, Morocco; 2 Department of Medical Oncology, Mohamed V Military Hospital, Rabat, Morocco; 3 Center pathology UN, Rabat, Morocco; 4 Department of Radiotherapy, National Institute of Oncology, Rabat, Morocco

## Abstract

**Background:**

The head and neck is the second most common region for the extra-nodal lymphomas after that of gastrointestinal tract. Approximately 2.5% of malignant lymphoma arises in the oral and para-oral region. In this paper we report two cases of early stage head and neck lymphoma which were managed successfully with chemotherapy and a review of the related literature.

**Cases presentation:**

The first case concerns a 48 years male patient having a diffuse large B-Cell lymphoma of the oropharynx at early bulky stage. This patient was managed successfully with 7 of Rituximab 375 mg/m^2^, Cyclophosphamide 750 mg/m^2 ^d1, Doxorubicine 50 mg/m^2 ^d1, Vincristine 1.4 mg/m^2 ^d1, and prednisone 50 mg/m^2 ^d1-5 (RCHOP) regimen. The second case concerns a 50 years female patient having the nasal natural killer (NK)/T-cell lymphoma of the left nasal pit at early stage. This case was managed successfully with 6 of Cyclophosphamide 750 mg/m^2 ^d1, Doxorubicine 50 mg/m^2 ^d1, Vincristine 1.4 mg/m^2 ^d1, and prednisone 50 mg/m^2 ^d1-5 (CHOP) regimen.

**Conclusion:**

These two cases highlight the important role of CHOP based chemotherapy for achieving successful treatment cure for patients having an early stage head and neck lymphoma.

## Introduction

Lymphomas are malignant neoplasms of the lymphocyte cell lines. They mainly involve lymph nodes, spleen and other non-haemopoietic tissues. They are mainly classified as either Hodgkin's or non-Hodgkin's lymphoma (NHL), and of either B-lymphocyte or T-lymphocyte origin. The head and neck is the second most common region for the extra-nodal lymphomas after that of gastrointestinal tract. Approximately 2.5% of malignant lymphoma arise in the oral and paraoral region, mainly in the form of Waldeyer's ring (ie, the tonsils and base, nasopharynx and base of the tongue) [1]. The diffuse large B-cell lymphoma (DLBCL) appeares to be the most common type of primary oral and paraoral NHL [1,2]. In this paper, we will present two cases of early stage head and neck lymphoma, the first with DLBCL of the oro-pharynx at early bulky stage and the second with nasal NK/T cell lymphoma of the left nasal pit at early stage. The two patients were managed successfully with CHOP based chemotherapy treatment.

## Case 1

**Figure 1:**
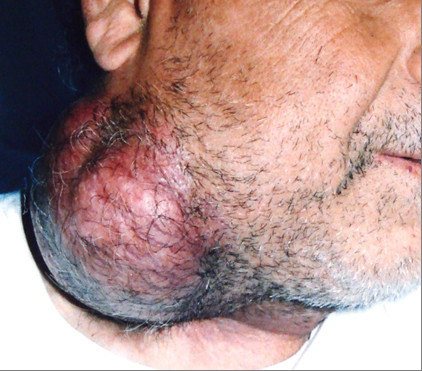
**Right cervical masse fixed, bulky, measured 13 × 10 cm in diameter**.

**Figure 2:**
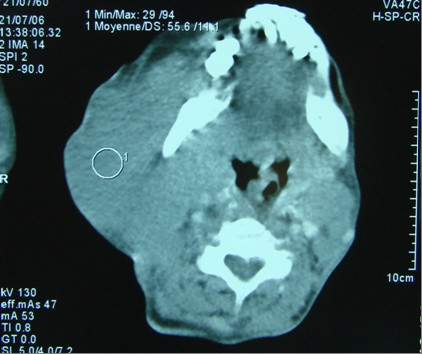
**Computed tomography of the head and neck shows the oropharyngeal process and a bulky cervical mass**.

**Figure 3:**
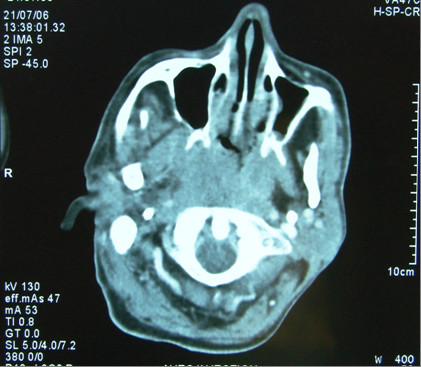
**Computed tomography of the head and neck shows the involvement of the nasopharynx by the oropharyngeal process**.

**Figure 4:**
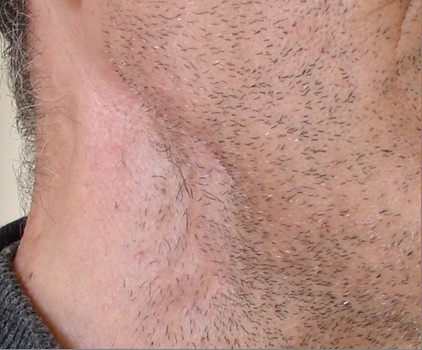
**The right cervical region was free from the disease 22 month after the end of chemotherapy**.

A 48 years old man, was admitted to the National Institute of Oncology hospital with enlarged cervical lymph nodes, dysphagia, dysphonia and having continuous weight loss for 5 months. The patient had an ECOG performance status equal to 2.0 [3]. A physical examination revealed a fixed cervical masse measuring 13 cm long and 10 cm large (figure 1). Otolaryngology examination showed an ulcerative-vegetative tumour on the right posterolateral wall of the oropharynx. The tumour spread to the soft palate ant hard palate filling partially the nasopharynx. Head and neck computed tomography scan showed a large tissular oropharyngeal tumour (Figure 2). The oropharyngeal mass invaded the nasopharynx (Figure 3). This process was associated by the infiltration of the tonsillar fossa and the parotid space by a bulky cervical mass (13 cm × 10 cm) growing on the right cervical region vast, starting from the submaxillary region up to the supra-clavicular region (Figure 2). The oropharyngeal biopsy was performed. Histological and immunohistochemistry studies showed diffuse large B-cell Lymphoma of the oropharynx according to the Revised European-American Classification of Lymphoid Neoplasms/World Health Organisation classification of lymphoid neoplasms (REAL/WHO). Most of the neoplasic cells were positive for CD-20 and for leucocyte common antigen (LCA) antibody. Computed tomography of the chest, abdomen and pelvis was normal. A bone marrow biopsy showed no abnormalities. The patient was staged IIEXB according to the Ann Arbor Staging system. The patient received 7 cycles of standard Rituximab 375 mg/m^2^, Cyclophosphamide 750 mg/m^2 ^d1, Doxorubicine 50 mg/m^2 ^d1, Vincristine 1.4 mg/m^2 ^d1, and prednisone 50 mg/m^2 ^d1-5 (RCHOP) regimen with complete response. He remained disease free, until now, 22 months after the end of chemotherapy (Figure 4).

## Case 2

**Figure 5:**
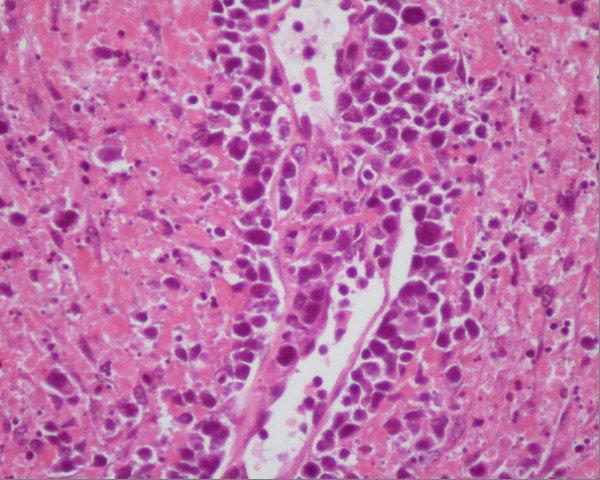
**NK/T-cell lymphoma, angiocentric infiltrate of small lymphocytes and atypical cells**.

**Figure 6:**
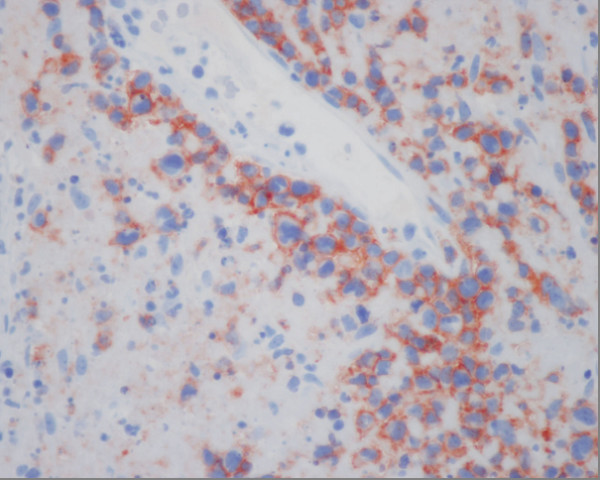
**Angiocentric marking by CD 56**.

A 50 years old female was admitted to the Oncology hospital. She had 5 months history of running nose (clear liquid at the beginning then becoming yellowish at the end). Evolution was marked by a foreign body sensation in the nasal passages without general signs. Naso-fibroscopy examination showed a process of the left nasal pit, inserted into the nasal septum. Histological and immunohistochemistry studies showed the signs of malignant non-Hodgkin lymphoma NK/T nasal type, with strong expression of CD3 and CD56 (figure 5) (Figure 6) (Figue 7). Head and neck computed tomography scan showed a tumour of the left nasal pit with a mass volume measuring 4 cm × 2 cm × 3 cm. Computed tomography of the chest, abdomen and pelvis was normal. A bone marrow biopsy showed no abnormalities. The patient was staged IE according to the Ann Arbor Staging system. The patient received 6 cycles of standard Cyclophosphamide 750 mg/m^2 ^d1, Doxorubicine 50 mg/m^2 ^d1, Vincristine 1.4 mg/m^2 ^d1, and prednisone 50 mg/m^2 ^d1-5 (CHOP) regimen. The response to the treatment was successful. The patient, 6 months after the end of chemotherapy, remains disease free. She is continuously followed by our group up to now.

**Figure 7:**
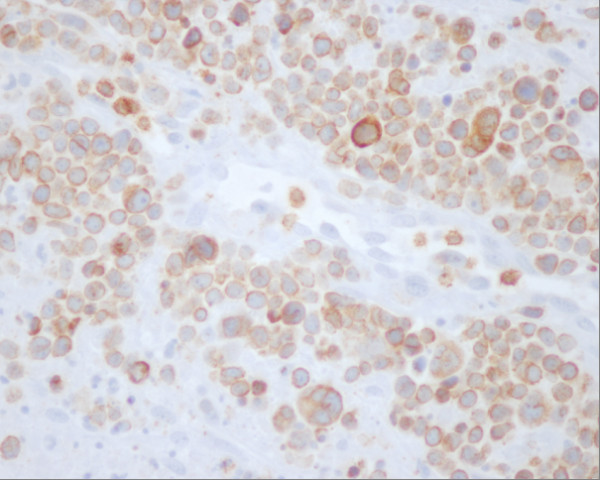
**Angiocentric marking by CD 3**.

## Discussion

The head and neck is the second most common region for the extra-nodal lymphomas. Approximately 2.5% of malignant lymphoma arises in the oral and paraoral region, mainly in the form of Waldeyer's ring (ie, the tonsils, nasopharynx and base of the tongue) [1].

The DLBCL is the most common type of primary oral and paraoral NHL [1,2]. Nasal non-Hodgkin's lymphoma is a rare clinical entity in western countries, but the disease is common in East Asian countries and Latin America [4]. In the revised European-American lymphoma and WHO classification, primary nasal and nasal type NK/T-cell lymphoma are recognized as distinct clinico-pathologic entities [4].

Several factors are known to increase NHL risk including the Epstein-Barr virus [5]. In one serie, primary oral and para-oral lymphoma was most commonly presented as a painless local mass that gradually increased in size with superficial ulceration. Most of the patients with a tonsillar NHL complained of dysphagia or sensation of a foreign body in the throat [6]. Our first patient presented cervical lymph nodes, dysphagia, dysphonia and weight loss with ulcerative-vegetative tumour (otolaryngology examination). Therefore, the symptoms were similar to those of a squamous cancer, which can only be differenciated by biopsy in the oropharynx.

The presence of positive staining for leukocyte common antigen (LCA) in histological specimen distinguishes malignant lymphomas from non-lymphoide neoplasms. Diffuse large B-cell lymphoma was most commonly positive for CD20 and CD79a and less commonly positive for germinal centre cell markers CD10 and BCL6. A small minority of cases showed a translocation between the BCL-2 gene on chromosome 18 and the IgH gene on chromosome 14, t(14;18) [1]. Nasal type NK/T-cell lymphoma arises as consequence of malignant transformation of NK-cells (NKC) which express markers CD 56+ (Neural cell adhesion molecule), TIA (T-cell intracellular antigen-A), and lack T-cell receptor (TCR) gene rearrangements, hence distinguishing the tumour from T-cell lymphomas [7,8]. The patient showed positive staining for CD3 and CD56. Most commonly, this tumour affects the nose and mid face although the disease can also arises on the skin, the gastro-intestinal tract, the testicle, the CNS, the lung, the salivary glands, the bone marrow, and the larynx [8].

Computed thomography of the head and neck, chest, abdomen and pelvis is the mainstay of staging for oropharyngeal lymphoma as well as other nodal lymphomas. Bone marrow biopsy is equally mandatory for staging. Concurrent positron emission tomography (PET) with 18F-fluorodeoxyglucose (FDG) and computed tomography (PET/CT) is a useful method for staging and assessment of therapeutic response [9].

In general, the standard treatment for patients with early stage diffuse large-cell lymphoma is chemotherapy (CT) followed by involved field radiotherapy (RT) [10,11].

Treatment recommendation for diffuse large B-cell lymphoma is generally identical for nodal and extra nodal diseases [12]. The use of chemotherapy is based on the principle that DLBCL of the head and neck must be considered a localised manifestation of systemic disease. Patients with stage I and II DLBCL (not bulky) usually do well with systemic chemotherapy followed by radiotherapy [1]. The prognosis for such patients is much better than that of patients with obvious systemic disease [13]. Patients with advanced-stage (bulky stage II, stage III and IV) must be treated by combined chemotherapy. Eight of RCHOP regimen should be considered as the standard treatment for patients with advanced stage DLBCL [14]. Our patient was staged IIXE bulky and was treated by 7 cycles of RCHOP regimen with good control of the disease until now (22 months after the end of chemotherapy).

Nasal NK/T cell lymphomas are rare, and the optimal treatment has still not been clearly established. The management of Nasal NK/T-cell lymphomas has been based largely on extrapolation from the experience with aggressive NHL. Ongoing studies has been limited to small institutional series, heterogeneous treatments and variable diagnostic criteria [10,11]. Overall, for patients with the early stage disease, chemotherapy (CT) followed by involved field radiotherapy (RT) is considered the standard treatment [9,10]. Our patient achieved complete and successful response after the end of 6 cycles of chemotherapy.

According to the international prognostic index (IPI) established for patients aged less than 60 years, the outcome of patients with extra nodal DLBCL is similar to that of patients with nodal DLBCL [12]. Nasal NK/T-cell lymphoma shows a more aggressive behaviour, poorer prognosis and frequent relapses.

## Conclusion

These two cases highlight the important role of CHOP based chemotherapy for achieving the cure for patients with localised stage diffuse large B-cell and T-cell lymphoma of the head and neck.

## Consent

The authors obtained written, informed consent from the patients for open access publication of this case report.

## Competing interests

The authors declare that they have no competing interests.

## Authors' contributions

IE and NI contributed equally to this work. All authors have made significant contributions by making diagnosis and intellectual input in the case and writing the manuscript.

## Acknowledgements

We sincerely thanks Pr Mohammed Ismaili for his assistance
